# *Escherichia coli* Producing Colibactin Triggers Premature and Transmissible Senescence in Mammalian Cells

**DOI:** 10.1371/journal.pone.0077157

**Published:** 2013-10-08

**Authors:** Thomas Secher, Ascel Samba-Louaka, Eric Oswald, Jean-Philippe Nougayrède

**Affiliations:** 1 INRA, USC 1360, Toulouse, France; 2 INSERM, UMR 1043, Toulouse, France; 3 CNRS, UMR 5282, Toulouse, France; 4 Université de Toulouse, UPS, Centre de Physiopathologie Toulouse Purpan (CPTP), Toulouse, France; 5 CHU Toulouse, Hôpital Purpan, Service de bactériologie-Hygiène, Toulouse, France; Boston University Medical School, United States of America

## Abstract

Cellular senescence is an irreversible state of proliferation arrest evoked by a myriad of stresses including oncogene activation, telomere shortening/dysfunction and genotoxic insults. It has been associated with tumor activation, immune suppression and aging, owing to the secretion of proinflammatory mediators. The bacterial genotoxin colibactin, encoded by the *pks* genomic island is frequently harboured by *Escherichia coli* strains of the B2 phylogenetic group. Mammalian cells exposed to live *pks+* bacteria exhibit DNA-double strand breaks (DSB) and undergo cell-cycle arrest and death. Here we show that cells that survive the acute bacterial infection with *pks*+ *E. coli* display hallmarks of cellular senescence: chronic DSB, prolonged cell-cycle arrest, enhanced senescence-associated β-galactosidase (SA-β-Gal) activity, expansion of promyelocytic leukemia nuclear foci and senescence-associated heterochromatin foci. This was accompanied by reactive oxygen species production and pro-inflammatory cytokines, chemokines and proteases secretion. These mediators were able to trigger DSB and enhanced SA-β-Gal activity in bystander recipient cells treated with conditioned medium from senescent cells. Furthermore, these senescent cells promoted the growth of human tumor cells. In conclusion, the present data demonstrated that the *E. coli* genotoxin colibactin induces cellular senescence and subsequently propel bystander genotoxic and oncogenic effects.

## Introduction

Cellular senescence has been defined by Hayflick and Moorhead as an irreversible state of cell-cycle arrest that is unresponsive to growth factors [1]. They observed that after a certain number of population doublings, proliferating mammalian cells spontaneously reach an irreversible cell-cycle arrest [1]. This was referred as replicative senescence and demonstrated as the results of DNA damage response (DDR) consecutive to telomere shortening [2]. However, senescence can also occur prematurely upon a myriad of cellular stresses without significant telomere erosion [3]. These stimuli include oxidative stress, ionizing/non ionizing radiations and DNA-damage inducing chemicals [3-5]. Whatever the stimuli, there are considerable evidences suggesting that most cases of stress-induced senescence result in accumulation of DNA damage and consequently induce premature senescence and aging [2,6,7].

Prominent senescence-associated characteristics are enlarged flat morphology [1] concomitant with senescence-associated beta-galactosidase (SA-β-Gal) expression [8], chronic activation of DDR signals [4,9], cyclin-dependent kinase inhibitors (CKI) p16^INK4a^ and/or p21^CIP1^ expression [10] orchestrating the formation of senescent-associated heterochromatin foci (SAHF) [11], and altered expression and secretion of numerous cytokines, growth factors and proteases with potent auto- and/or paracrine activity [12] termed senescence-associated secretory profile (SASP).

We recently identified in certain *E. coli* strains of the phylogenetic group B2 a genomic island named “*pks* island” [13,14]. This cluster of genes encodes the production of a putative hybrid peptide-polyketide genotoxin, named colibactin, that induces DNA-double strand breaks (DSB) both *in vitro* in mammalian cell lines [13] and *in vivo* in enterocytes [15]. *E. coli* are pioneer bacteria colonizing massively the gastrointestinal tract of mammals within few days after birth and becoming the predominant facultative anaerobic bacteria in the adult microbiota [16,17]. Recent studies showed that the prevalence of *E. coli* strains of the phylogenetic group B2 is increasing in human microbiota from industrialized countries [17,18]. Up to 50% of *E. coli* strains isolated from children faeces belong to the B2 phylogenetic group [17,19]. Epidemiological surveys showed that up to 34% of these commensal B2 strains carried the *pks* island [13,20,21]. This high prevalence prompted us to examine the consequences of colibactin-inflicted damage on mammalian cells.

Acute infection with *pks*+ *E. coli* causes massive DSB followed by DDR activation, cell cycle arrest and apoptosis [13]. This DNA damage can be repaired in surviving cells by the DDR machinery and then the cells resume the cell cycle [15]. However we previously showed that some of the DNA damage persists and eventually triggers chronic chromosomal aberrations and instability [15]. Based on the observations that the DDR is activated upon senescence and may propel genomic instability and telomere shortening [7,22] and our previous findings suggesting that colibactin induces persistent DNA damage [15], we asked whether it could induce cellular senescence.

We report here that infection of human cells with *pks*+ *E. coli* can induce persistent DNA damage together with an irreversible cell-cycle arrest. The cells exhibited typical features of cellular senescence with the expression of SA-β-Gal and of CKI, formation of promyelocytic leukemia (PML) nuclear bodies and of SAHF. This was related with cellular ROS and SASP production. This SASP exerted paracrine effects in recipient naive cells, with bystander induction of γH2AX foci and SA-β-Gal, and promoted the proliferation of cancer cells. Collectively, these data indicate that *pks*+ *E. coli* strains can induce cellular senescence that could facilitate carcinogenesis and accelerate aging.

## Experimental Procedures

### Bacterial strains and culture

For the *in vitro* assays, we used the previously described *E. coli* strain DH10B hosting a BAC bearing the *pks* island (*E. coli pks*+) that produces colibactin and the DH10B hosting the vector only (*E. coli pks*−) [13]. For the infection assay, bacteria were cultured overnight in LB broth at 37 °C with shaking. The next day, this culture was diluted 1:100 in interaction medium (IM), consisting of DMEM buffered with 25 mM Hepes and supplemented with 10% Fetal calf serum (FCS, Eurobio). This preactivated culture was incubated at 37 °C with shaking until an optical density of 0.2-0.4 at 600nm was obtained. Then the bacteria were added to the cells.

### Cell culture

Nontransformed human lung fibroblast (IMR-90; ATCC CRL-186), nontransformed rat intestinal epithelial cells (IEC-6; ATCC CRL-1592), human lung adenocarcinoma epithelial cell (A549; ATCC CCL-185) and human colon carcinoma cell (HCT116 p53^-/-^, obtained from Dr Bert Volgenstein) were cultured in DMEM 10% FCS, 80 μg/mL gentamicin, 1% non essential amino acid (Invitrogen), and supplemented with 4 μg/mL insulin (Sigma) for IEC-6 cells. All cell lines were grown at 37 °C in a 5% CO_2_ humidified atmosphere and split regularly to maintain exponential growth. A fresh culture was started from a liquid nitrogen stock every 12 passages. The cell lines were confirmed free from mycoplasma contamination by PCR.

### In vitro infection assay

Cells (~75% confluent) were washed four times with warm HBSS (Invitrogen) and incubated in IM for 2 hours before infection. The bacteria were then added according to a given multiplicity of infection (MOI, number of bacteria per cell at the onset of the infection). After 4 hours of infection at 37°C in a 5% CO_2_ atmosphere, cells were washed four to six times with warm HBSS and then incubated in cell-culture medium supplemented with 200 µg/mL of gentamicin. The culture medium was changed the next day.

### Cell cycle, proliferation, mitochondrial dysfunction and ROS production flow cytometry analyses

Cells were collected by trypsin treatment. For cell-cycle analysis, cells were fixed overnight in 70% ethanol at −20 °C, then treated with PBS 0.1% Triton X-100 for 5 min, washed and resuspended in PBS containing 15 μg/mL propidium iodide (Sigma) and 100 μg/mL RNase (Fermentas). For proliferation analysis, cells were processed with the Click-iT EdU Flow Cytometry Assay Kit (Invitrogen), according to the manufacturer’s instructions. For mitochondrial dysfunction analysis, live cells were treated 10 min at 37°C with 5µM Mitosox (Invitrogen) prepared in HBSS and then washed four times in HBSS. For ROS production analysis, live cells were treated 20 min at 37°C with 10 µM H2DCFDA (Invitrogen) prepared in HBSS and then washed four times in HBSS. Flow cytometry analyses were done with a FACS Calibur flow cytometer (Becton Dickinson) and FlowJo software (Tree Star). For cell cycle analysis, aggregates were removed according to the FL2W/FL2A profile during acquisition. At least 2x10^4^ cells were acquired per sample.

### Immunofluorescence analyses

Cells were grown and infected on LabTech slides (Falcon). For γH2AX, PML and SAHF analysis, cells were fixed with PBS 4% formaldehyde for 10 min, permeabilized in PBS 0.25% Triton X-100 for 5 min, and blocked with PBS 0.1% Tween and 5% normal goat serum for 30 min. Primary antibodies mouse anti-γH2AX (Millipore, clone JBW301), rabbit anti-PML (Santacruz, clone H-238) and rabbit anti trimethyl-histone 3 (Millipore, Lys9) were diluted 1:400, 1:100, 1:100 respectively in blocking solution and incubated overnight at 4 °C. FITC-conjugated affinity-purified secondary goat antibodies (Zymed) were diluted 1:500 in blocking solution and incubated for 1h. DNA was stained for 5 min with TO-PRO-3 (Molecular Probes) or with DAPI (Vector) for SAHF analysis. Slides were mounted in VectaShield containing DAPI (Vector). For Mitosox and H2DCFDA analysis, live cells were treated respectively with 5µM or 10µM of each probe for 10 or 20min and washed four times with HBSS. Slides were mounted in warm HBSS and analysed during the next hour. Images were acquired with an Olympus IX70 laser scanning confocal microscope using a 60X PlanApo NA=1.6 objective. Optical sections (z=0.35 µm) were captured in sequential mode, with the Fluoview software FV500.

### Western Blot and In-Cell Western analyses

Cell lysates were prepared in Laemmli loading buffer, sonicated for 2s to shear DNA and then heated at 70°C for 10min. Protein samples were resolved on 4-12% NuPage gradient gels (Invitrogen) and blotted on PDVF membranes. Membranes were blocked in TBST (10 mM Tris pH 7.8, 150 mM NaCl, 0.1% Tween 20) supplemented with 10% non-fat dry milk, then probed with primary antibodies (0.5 mg/ml) in TBST supplemented with 5% non-fat dry milk. Primary antibodies used were mouse monoclonal anti-actin (MP Biomedicals), mouse monoclonal anti-p21/CIP1 (Cell Signaling Technology, DCS60) and rabbit polyclonal anti-p16 (Santa cruz Biotechnology, sc-468). Bound antibodies were visualized with horseradish peroxidase-conjugated secondary antibodies. Acquisitions were performed with a Molecular Imager ChemiDoc XRS System (Bio-Rad). Proteins were quantified with Quantity One software (Bio-Rad) and normalized with actin level.

The In-Cell Western procedure was performed as previously described [23]. Briefly, the cells were fixed with 4% paraformaldehyde, permeabilized with 0.2% Triton X-100, blocked with MAX block Blocking medium (Active Motif, Belgium) supplemented with phosphatase inhibitors PhosSTOP (Roche) and incubated overnight with rabbit monoclonal anti γH2AX antibodies (Cell Signaling) (1:200). An infrared fluorescent secondary antibody (IRDyeTM 800CW, Rockland) was then applied (1:500) together with RedDot2 (Biotium) (1:500) for DNA labelling. DNA and γH2AX were quantified using an Odyssey Infrared Imaging Scanner (Li-Cor ScienceTec, Les Ulis, France) at 680 nm and 800 nm respectively. Fluorescent units for γH2AX relative to DNA content were determined using the Odyssey software.

### Senescence associated-β-galactosidase staining

SA-β-Gal staining was performed according to Dimri et al [8]. At least 200 cells were counted. Images were acquired with a Leica DMRB microscope, DFC300 Fx camera and Leica LAS software.

### Conditioned medium (CM), SASP analysis, bystander and insert experiments

Cells were infected as above, grown in complete cell cultured medium for 3, 6, 9 or 14 days then washed two times with PBS and further incubated 24 hours in serum-free culture medium. Culture supernatants were then harvested, clarified from debris by centrifugation, filtered on 0.22 µm and stored in aliquots at -80°C. These CM were analysed for IL-6, IL-8, MCP-1 and MMP-3 expression using magnetic Bio-Plex Human Cytokine and MMP multiplex assay (Biorad) according to the manufacturer’s instructions. Data acquisition was made using a Luminex 200 analyser (Luminex). For ELISA experiments, cells were infected in a 96-well plate with a final volume of 100 µL per well for 1 or 3 days. Culture supernatants were then harvested, clarified from debris by centrifugation, filtered on 0.22 µm and stored in aliquots at -80°C. These CM were analysed for IL-6 and MMP3 using ELISA kit (Human Duoset Kit, R&D).

For bystander experiments, naive cells (~75% confluent) were washed four times with warm HBSS (Invitrogen) and incubated with CM for 3 days. Cells were subsequently analysed for γH2AX and SA-β-Gal expression. For the insert assay, IMR-90 cells were grown on 24-well Transwells (Corning) for 24 h and infected with *E. coli* strains for 4 h. Three days after the infection, the Transwells were transferred over a subconfluent layer of naive IEC-6 cells or over 5000 HCT116 p53^-/-^ or A549 cells. To test the role of ROS in bystander damage n-acetylcysteine (Sigma) was added to the medium at 1mM. The IEC-6 cells were analysed for γH2AX by In-Cell Western after 24h. A549 and HCT116 p53^-/-^ cells were incubated for 5 days and assayed for proliferation using an MTT assay (Promega).

### Co-culture and layered soft agar colony formation experiments

15 to 30x10^4^ infected IMR-90 cells were grown to confluence in 6-well plates in DMEM 10% FCS 80 μg/mL gentamicin. Cells were washed twice in PBS and then 5000 A549 cells were added in each well and the co-cultures were further incubated for 15 days in DMEM 1% FCS culture medium. The co-cultures were washed three times with PBS, fixed 10 min with PBS 4% formaldehyde and stained with 1% Rhodanile blue, which preferentially stains epithelial cell colonies [24]. Images were acquired with an Epson Perfection 3170 scanner and imported into Adobe Photoshop. Rhodanile Blue staining area in each well was quantified in the green channel using NIH Image-J software.

For the layered soft agar colony assay, IMR-90 cells were infected and grown to confluence in 6-well plates as above. After 2 washes with PBS, 1 ml of 0.5% agar in DMEM 1% FCS at 42°C was added on top of the fibroblasts to form a base layer. After the agar was solidified, 5000 HCT116 p53^-/-^ cells suspended in 1 ml of 0.35% agar in DMEM 1% FCS at 42°C were added over the base layer. The plates were incubated 10 days in a humidified 37°C 5% CO_2_ incubator. The HCT116 p53^-/-^ colonies were then microphotographed and counted (size larger than 225 µm) in 5-7 random fields using an Olympus CKX31 inverted phase-contrast epi-illuminated microscope. For further quantification, the upper cancer cell layer was aspirated gently and transferred onto a 6-well plate and stained 16 h with MTT (Promega). Images of the plates were acquired with an Epson Perfection 3170 scanner and formazan-stained cellular colonies were counted using NIH Image-J software. The cell proliferation was also quantified following overnight solubilisation of the formazan crystals in the solubilisation/stop solution (CellTiter96 assay, Promega) and recording of absorbance at 570 nm.

### Statistical analyses

Statistical evaluation of differences between the experimental groups was determined by using one-way analysis of variance (ANOVA) followed by a Bonferroni post-test (which allows comparison of all pairs of groups). All tests were performed with GraphPad Prism 5.03 (GraphPad Software Inc., San Diego, CA, USA). All data are presented as mean+standard error of the mean (SEM). A *p* value <0.05 was considered significant.

## Results

### Infection with colibactin producing *E. coli* induces persistent DNA damage, irreversible cell cycle arrest together with increased CDK inhibitors expression

**Figure 1:**
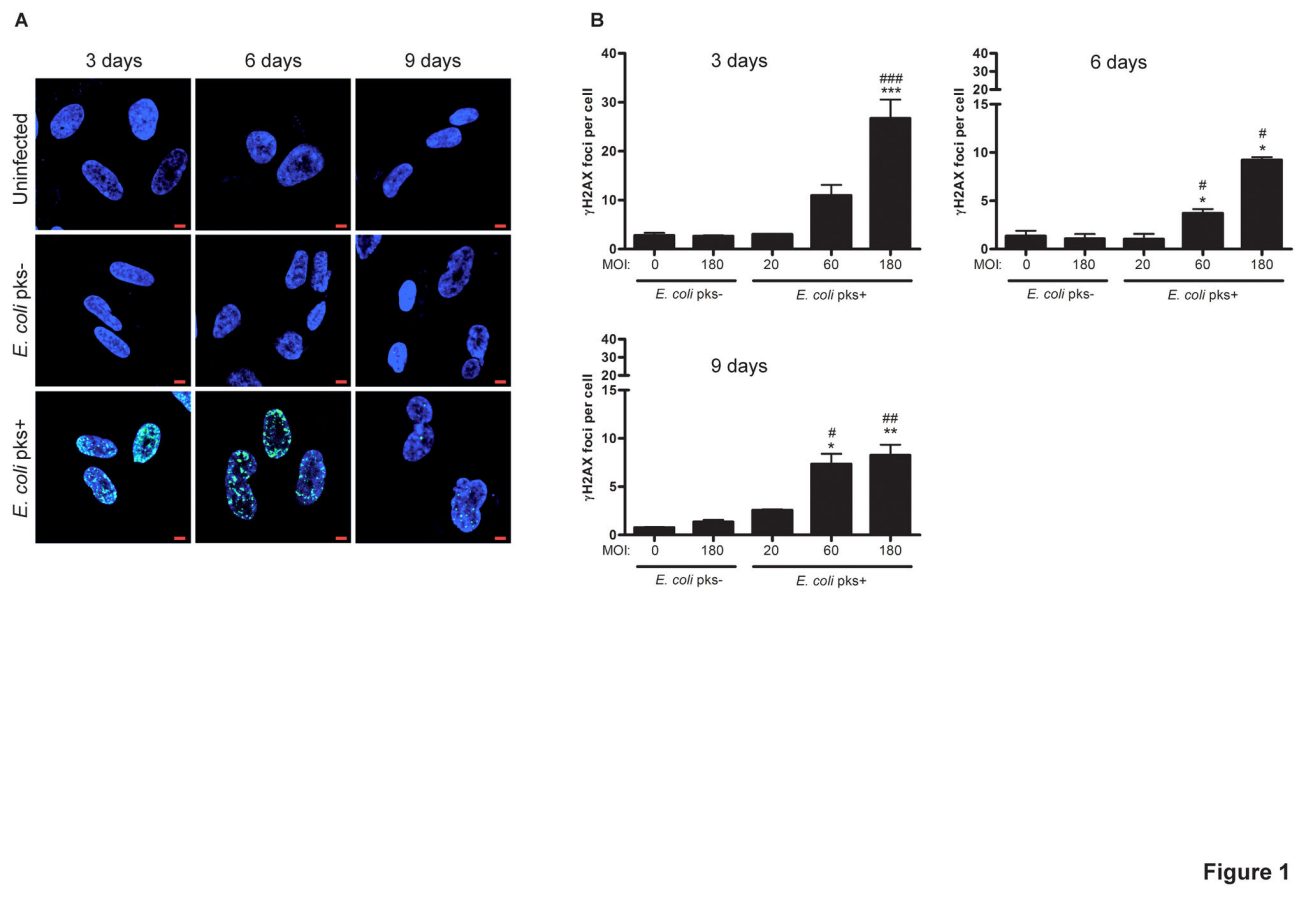
Persistent γH2AX foci in IMR-90 cells infected with *pks*+ *E. coli.* Non-transformed human IMR-90 cells were infected for 4h with live *pks*+ or *pks*- *E*. *coli* with a multiplicity of infection (MOI) of 20, 60 and 180 bacteria per cell or left uninfected. At the end of the infection, the cells were washed and further grown in medium supplemented with gentamicin. (A) Cells were examined for DNA (blue) and γH2AX (green) 3, 6 or 9 days after infection. Pictures of uninfected and MOI 180 -infected cells are shown, scale bars = 10µm. (B) The numbers of γH2AX foci per cell were quantified by a blinded observer in 30-100 nuclei for each condition in two independent experiments. Results represent the mean and standard error of the mean (SEM). Statistical significance was examined by one-way ANOVA with Bonferroni’s multiple comparison test; *P<0.05, **P<0.01, ***P<0.001 comparing infected and uninfected groups; #P<0.05, # #P<0.01, # # #P<0.001 comparing *pks*+ and *pks*- groups.

Based on our previous results showing that infection with *E. coli* producing colibactin causes acute DSB with the induction of the DDR pathways [13] but that low-dose infection induces transient DNA damage followed by repair [15], we first analysed over an extended period of time and with acute infectious dose, whether colibactin still induces persistent DNA damage. To do so, we infected non-transformed human IMR-90 fibroblasts, classically used to study of cellular senescence, with a *pks*+ or *pks*- *E. coli* strain at various multiplicity of infection (MOI) and followed the presence of DSB detected as nuclear foci positive for the phosphorylated form of the histone H2AX (γH2AX) [25]. We found a significant increase in the number of γH2AX foci shortly after infection as previously described [13] but also a significant prolonged persistence of γH2AX foci, 3, 6 and 9 days after infection with *pks*+ *E. coli* (Figure 1A). The number of foci increased with the *pks*+ *E. coli* MOI, and gradually decreased with time, remaining significantly different from controls (Figure 1B).

**Figure 2:**
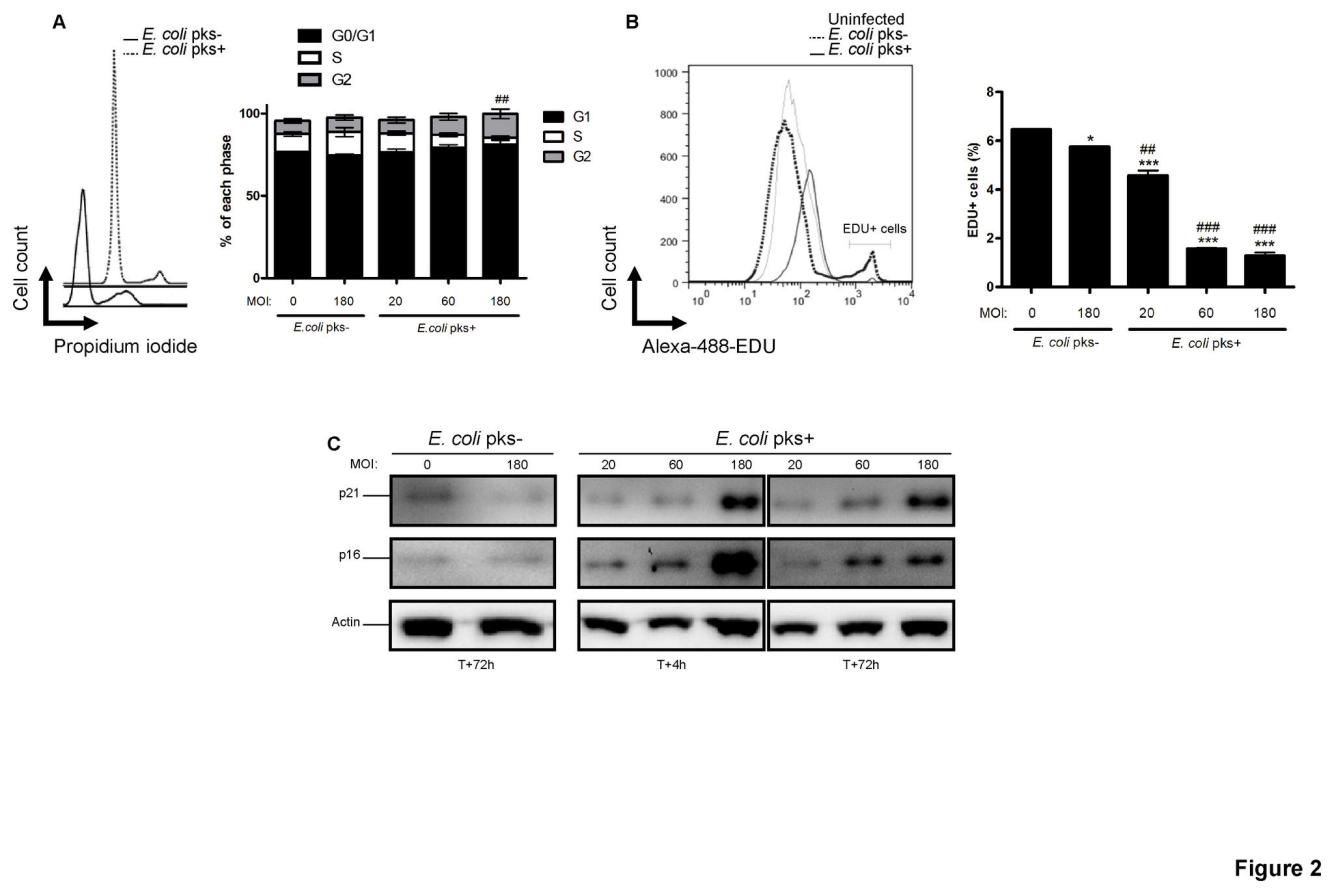
Permanent cell-cycle arrest and increased cell-cycle inhibitors expression in IMR-90 cells infected with *pks*+ *E. coli.* (A) Cell-cycle distribution was analyzed by flow cytometry 3 days after infection. (B) 3 days after the infection the cells were incubated with EdU for 2 hours and its incorporation in S-phase cells was analyzed by flow cytometry. (C) Western blot analysis of p21 and p16 protein content in IMR-90 cells 4h, 1 and 6 days after infection. Actin was probed as a protein loading control. Results represent the mean and SEM of two independent experiments, one-way ANOVA with Bonferroni’s multiple comparison test; *P<0.05, ***P<0.001 comparing infected and uninfected groups; # #P<0.01, # # #P<0.001 comparing *pks*+ and *pks*- groups.

We next examined the cell cycle of infected IMR-90 cells. We observed with *pks*+ *E. coli* a MOI-dependent decrease of cells in S-phase while G1- and G2-phases increased as compared to *pks*- *E. coli* or uninfected IMR-90 cells (Figure 2A). The lack of replication phase was confirmed by the analysis of EdU incorporation where less than 2% of the cells infected with *pks*+ *E. coli* at MOI 60 or 180 incorporated EdU in contrast to the control cell population with more than 6% of incorporating cells (Figure 2B).

The state of permanent replicative arrest that define cellular senescence [1] is governed by the induction of the CKI p21^CIP1^ or p16^INK4^ [10]. Consistent with the prolonged cell-cycle arrest following infection with *pks*+ *E. coli*, we observed dose-dependent elevated levels of the CKI p21^CIP1^ and p16^INK4^, rapidly after the infection (4h) and that were maintained 3 days after the infection (Figure 2C). We thus confirmed that colibactin-induced cell-cycle arrest was related to the induction of the classical senescence pathway that encompasses the activation of the CKI p21^CIP1^ and p16^INK4^.

### Infection with colibactin producing *E. coli* induces senescence-associated β-galactosidase expression

**Figure 3:**
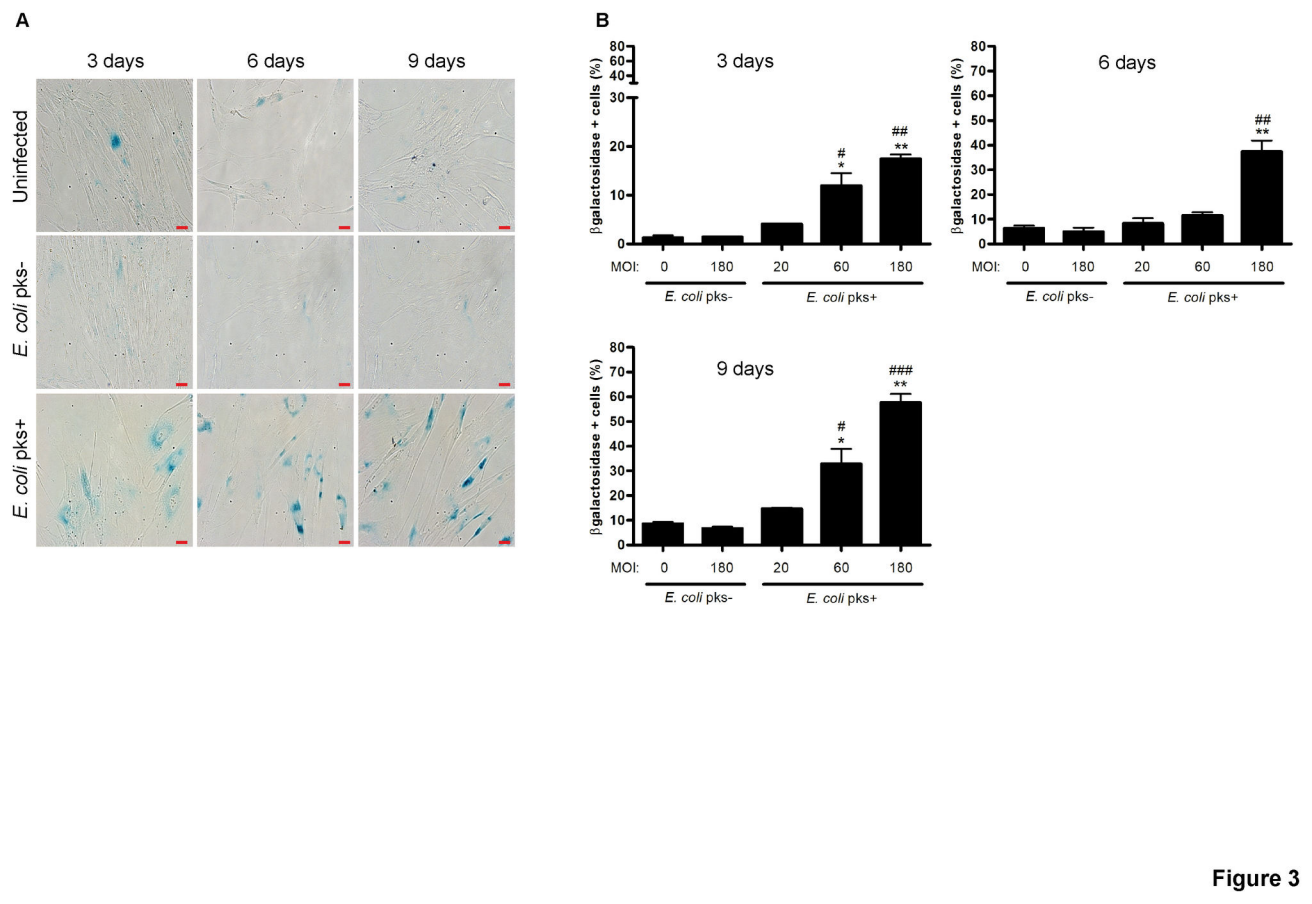
Increased senescence associated (SA)-β-galactosidase activity in IMR-90 cells infected with *pks*+ *E. coli.* 3, 6 or 9 days after infection, IMR90 cells were fixed with formaldehyde 4% and then stained with the galactosidase chromogenic substrate X-gal at pH 6 for 24h. Cells were examined for perinuclear blue staining by light microscopy at x20 magnification (A), and these SA-β-Gal positive cells (100-200 cells per condition) were quantified by a blinded observer (B). Results represent the mean and SEM of three independent experiments, one-way ANOVA with Bonferroni’s multiple comparison test; *P<0.05, **P<0.01 comparing infected and uninfected groups; #P<0.05, # #P<0.01, # # #P<0.001 comparing *pks*+ and *pks*- groups.

Persistent γH2AX foci, irreversible cell cycle arrest and recruitment of the p21^CIP1^ and p16^INK4^ CKI strongly suggested that colibactin induced cellular senescence. Based on these observations together with the enlarged cellular shape (termed megalocytosis) induced upon *pks*+ *E. coli* infection [13], we next assessed whether a well-known marker associated with cellular senescence, SA-β-Gal, was induced. SA-β-Gal was found significantly enhanced in IMR-90 cells infected with *pks*+ *E. coli* at MOI 60 or 180 as compared to *pks*- *E. coli* or uninfected cells (Figure 3A). The proportion of SA-β-Gal+ cells increased with the MOI and in time (Figure 3B). Increased SA-β-Gal was also found in non-transformed intestinal epithelial IEC-6 cells infected with *pks*+ *E. coli* (Figure S1C). These data confirmed that infection with *E. coli* producing colibactin elicits hallmarks of cellular senescence.

### Infection with colibactin producing *E. coli* promotes SAHF and PML-Nuclear Bodies (PML-NBs) formation

**Figure 4:**
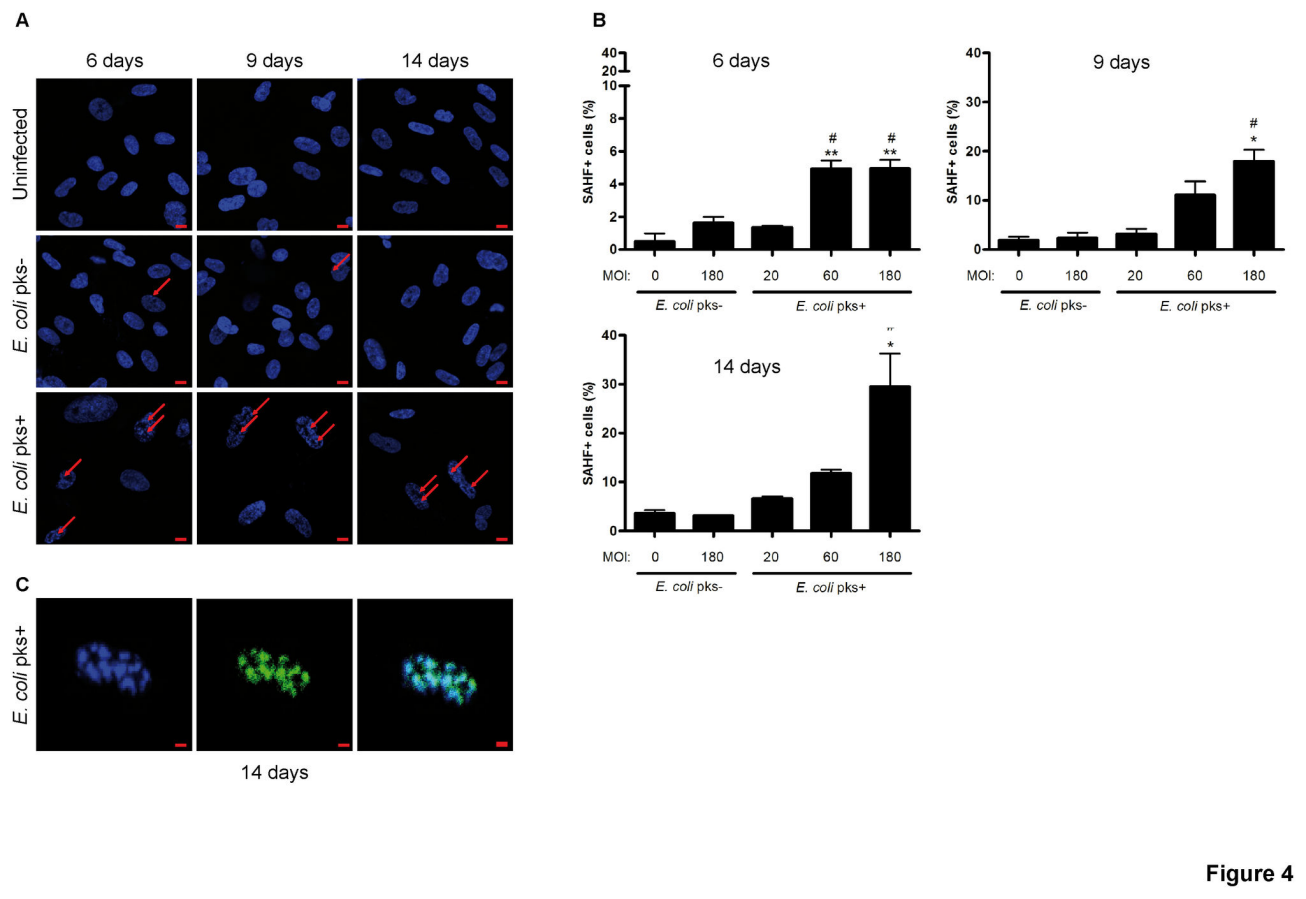
Accumulation of heterochromatin foci in IMR-90 cells infected with *pks*+ *E. coli.* (A) Cells were stained with DAPI 6, 9 or 14 days after infection and examined for formation of heterochromatin foci (arrows) by confocal microscopy. Pictures of uninfected and MOI 180 –infected cells are shown, scale bars = 10µm. (B) Cells were stained with DAPI (blue) and with antibodies against H3K9me3 (green) 14 days after infection and examined by confocal microscopy, scale bar = 50µm. (C) Heterochromatin foci-positive cells (60-100 nuclei for each condition) were counted by a blinded observer. Results represent the mean and SEM of three independent experiments, one-way ANOVA with Bonferroni’s multiple comparison test; *P<0.05, **P<0.01 comparing infected and uninfected groups; #P<0.05 comparing *pks*+ and *pks*- groups.

Cellular senescence has been associated with altered chromatin structure, manifested by the formation of typical SAHF which have been postulated to be involved notably in gene silencing responsible for cell-cycle arrest [11]. Thus, we examined the chromatin of IMR-90 cells at several days after infection. In *pks*+ *E. coli* -infected cells, DAPI stained nuclear DNA frequently exhibited punctate patterns (Figure 4A). SAHF formation involves numerous proteins including histone chaperones HIRA and Asf1 [26], heterochromatin protein HP1, high-mobility group A (HMGA) [11] proteins and a typical protein of transcriptionally silent heterochromatin such as lysine9-trimethylated histone H3 (H3Me) [11]. Indeed, we observed a clear correlation between SAHF and H3Me foci (Figure 4C). Quantification of these SAHF revealed an increased formation in a time and MOI-dependent manner following infection with *pks*+ *E. coli* as compared to *pks*- *E. coli* or uninfected cells (Figure 4B).

**Figure 5:**
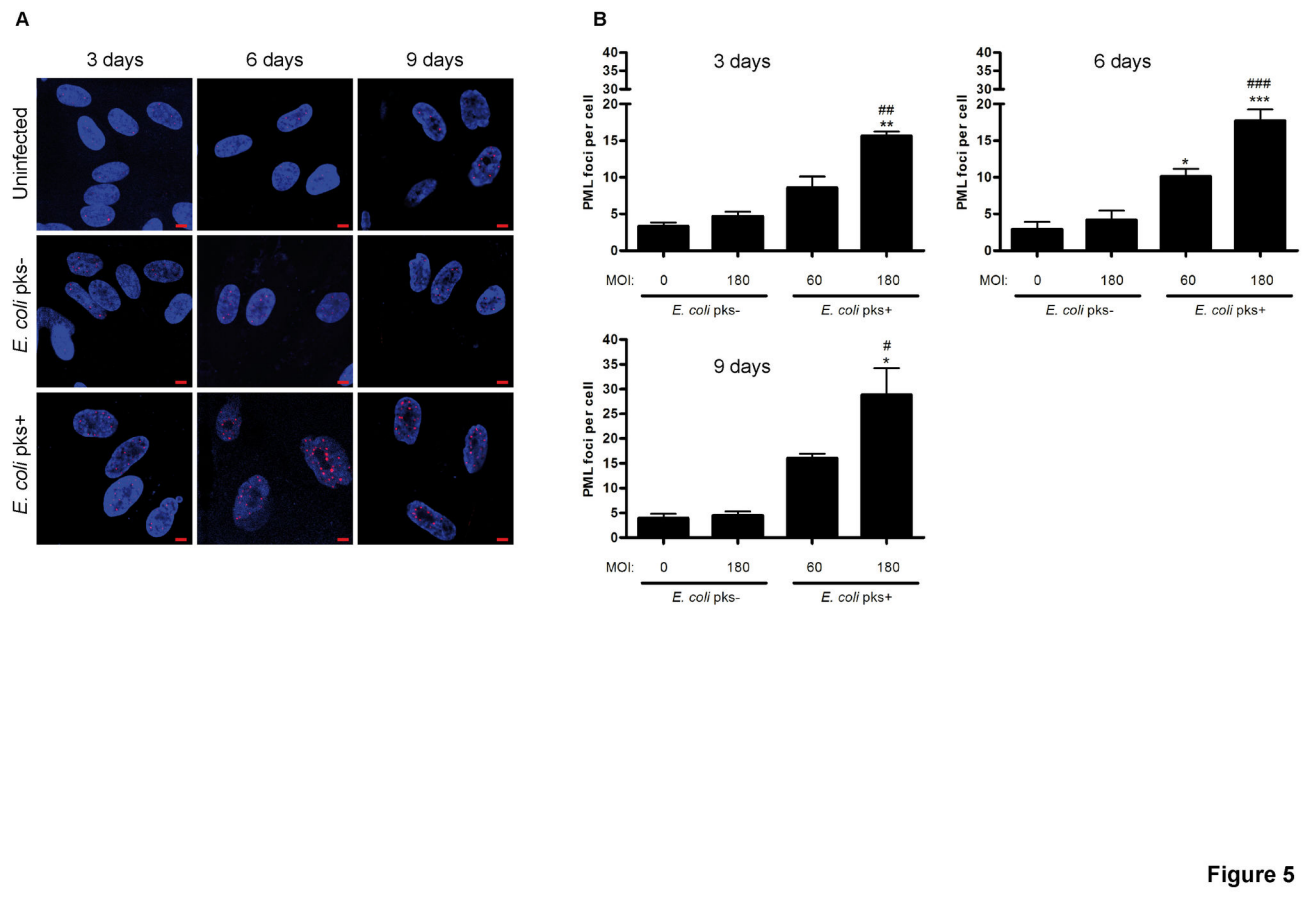
Increased PML nuclear bodies in IMR-90 cells infected with *pks*+ *E. coli.* (A) Cells were examined for DNA (blue) and PML protein (red) 3, 6 or 9 days after infection. Pictures of uninfected and MOI 180-infected cells are shown, scale bars = 10µm. (B) PML foci in 30-100 nuclei for each condition were counted by a blinded observer. Results represent the mean and SEM of three independent experiments, one-way ANOVA with Bonferroni’s multiple comparison test; *P<0.05, **P<0.01, ***P<0.001 comparing infected and uninfected groups; #P<0.05, # #P<0.01, # # #P<0.001 comparing *pks*+ and *pks*- groups.

Promyelocytic leukemia nuclear bodies (PML-NBs) are multicomponent subnuclear organelles that are involved in a range of host cell functions in response to stress including genotoxic stress [27] and that have been clearly associated with DNA damage [28,29]. Because PML-NBs act as general sensors of genomic damage [30], regulates cellular senescence [29,31] and are thought to participate in SAHF formation [26], we investigated whether PML-NBs could be affected following infection by *pks*+ *E. coli*. Indeed, we observed a significant time and MOI-dependent increase of PML foci in cells infected with *pks*+ *E. coli* as compared to controls (Figure 5A and B). Increased numbers of PML-NBs were also observed in intestinal epithelial IEC-6 cells infected with *pks*+ *E. coli* (Figure S1A and B). Hence the pro-senescent phenotype accompanied with the persistent DNA- damage induced by *pks*+ *E. coli* infection is linked with enhanced SAHF and PML-NBs formation.

### Colibactin-induced senescence is associated with intracellular and mitochondrial ROS production

**Figure 6:**
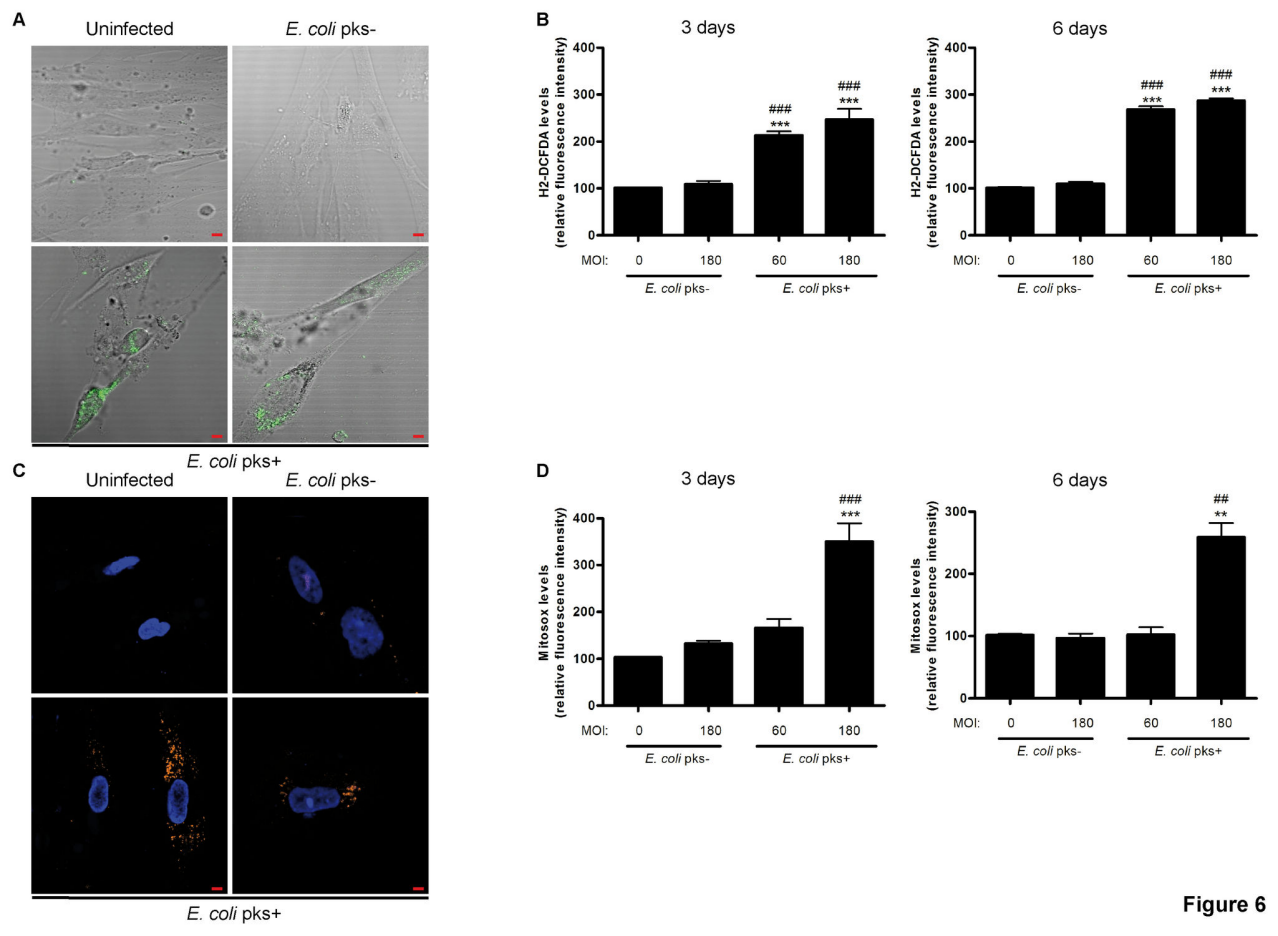
Increased intracellular and mitochondrial ROS production in IMR-90 cells infected with *pks*+ *E. coli.* (A) Cells were examined for intracellular ROS with the ROS sensor dihydro-dichloro-fluorescein diacetate (H2-DCFDA) (green). Pictures of uninfected and MOI 180-infected cells (at 3 days) are shown, scale bars = 10µm. (B) Mean H2-DCFDA fluorescence intensity was quantified by flow cytometry in 2x10^4^ cells, 3 and 6 days after infection. (C) Cells were examined for mitochondrial superoxide production with the mitochondrial superoxide probe MitoSOX (orange) and for DNA (blue). Pictures from uninfected and MOI 180-infected cells (at 3 days) are shown, scale bars = 10µm. (D) Mean MitoSOX fluorescence intensity was quantified by flow cytometry 3 or 6 days after infection. Results in panels B and D represent the mean and SEM of three independent experiments, one-way ANOVA with Bonferroni’s multiple comparison test; **P<0.01, ***P<0.001 comparing infected and uninfected groups; # #P<0.01, # # #P<0.001 comparing *pks*+ and *pks*- groups.

Several years before Hayflick and Moorhead defined the main feature of cellular senescence, Harman [32] proposed the free radical theory of aging, implicating reactive oxygen species (ROS) in the aging process. This theory was confirmed four decades later when several teams discovered that mild oxidative stress induces premature senescence in a wide variety of cell types [33,34]. We assessed whether colibactin producing *E. coli* infection could induce cellular ROS production. While we did not detect any difference of ROS production at early time points after infection, we observed in *pks*+ *E. coli* infected cells a significant augmentation of H2-DCFDA fluorescence (a sensor of hydroxyl, peroxyl radicals and peroxide hydrogen production) 3 and 6 days after the infection, when the phenotype of cellular senescence was clearly established (Figure 6A and B). Mitochondria are main sources of free radicals and the sites at which ROS are continuously produced. Electron leakage from the mitochondrial electron-transport chain involves up to 1-2% of healthy cells, while increasing in stress situations resulting in substantial increased ROS production [35]. Therefore, we investigated the effect of *pks*+ *E. coli* infection on the production of free radicals by mitochondria. We observed a time dependent increase in the proportion of Mitosox positive cells upon infection with *pks*+ *E. coli*, which indicates a selective superoxide production by mitochondria, as compared to *pks*- *E. coli* or uninfected cells (Figure 6C and D). Thus, the infection with *pks*+ *E. coli* is accompanied with a late persistent intracellular and mitochondrial ROS production.

### Colibactin-induced senescent cells exhibit enhanced pro-inflammatory mediator secretion

**Figure 7:**
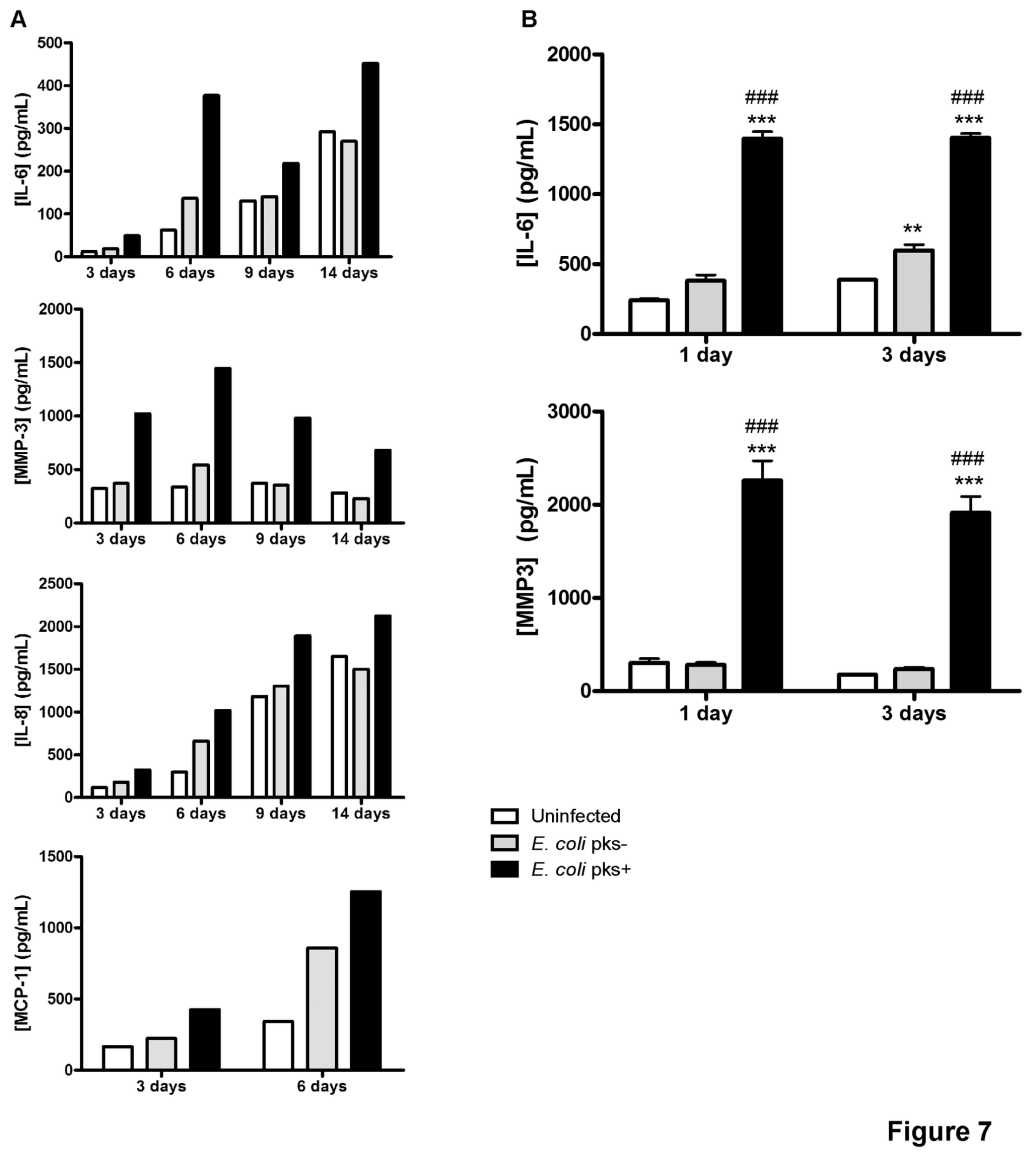
Induction of a senescence-associated secretory phenotype (SASP) in IMR-90 cells following infection with *pks*+ *E. coli.* IMR-90 cells were infected as before and incubated for 1-14 days. Conditioned cell culture media were prepared by incubating each culture in serum-free medium for 24 h. MMP-3, IL-6, IL-8 and MCP-1 secretion was quantified by Bio-Plex in one experiment (A) and by ELISA in two independent experiments (B) ; Results represent the mean and SEM, one-way ANOVA with Bonferroni’s multiple comparison test; **P<0.01, ***P<0.001 comparing infected and uninfected groups; # # #P<0.001 comparing *pks*+ and *pks*- groups.

Recent studies have shown that cellular senescence is accompanied by a striking increase in the secretion of 40–80 factors forming the so-called senescence associated secretory profile (SASP) that participate in intercellular signalling [36]. Using a multiplex assay, we analyzed the protein levels of interleukin (IL)-6, IL-8, monocyte chemotactic protein (MCP)-1 and matrix metalloprotein (MMP)-3 in the supernatant of infected IMR-90 cells at several times post-infection. Notably, we observed a significant increase of MMP-3 and IL-6 levels while IL-8 and MCP-1 levels were barely increased in the supernatant of cells infected with *pks*+ *E. coli* as compared to controls (Figure 7A). We confirmed these results in a second set of experiments where the secretion of proteins in the supernatant was concentrated using a volume of 100µL of culture medium in 96-well plate. We then measured the production of IL-6 and MMP-3, one and three days post-infection. We observed a sharp increase of IL-6 and MMP-3 in the supernatant of cells infected with *pks*+ *E. coli* as compared to controls (Figure 7B). Hence, we observed that cellular senescence response triggered by the infection with *pks*+ *E. coli* is associated with an acute and prolonged production of two hallmark components of the SASP.

### Colibactin-induced senescence is transmissible and evokes “bystander” senescence

**Figure 8:**
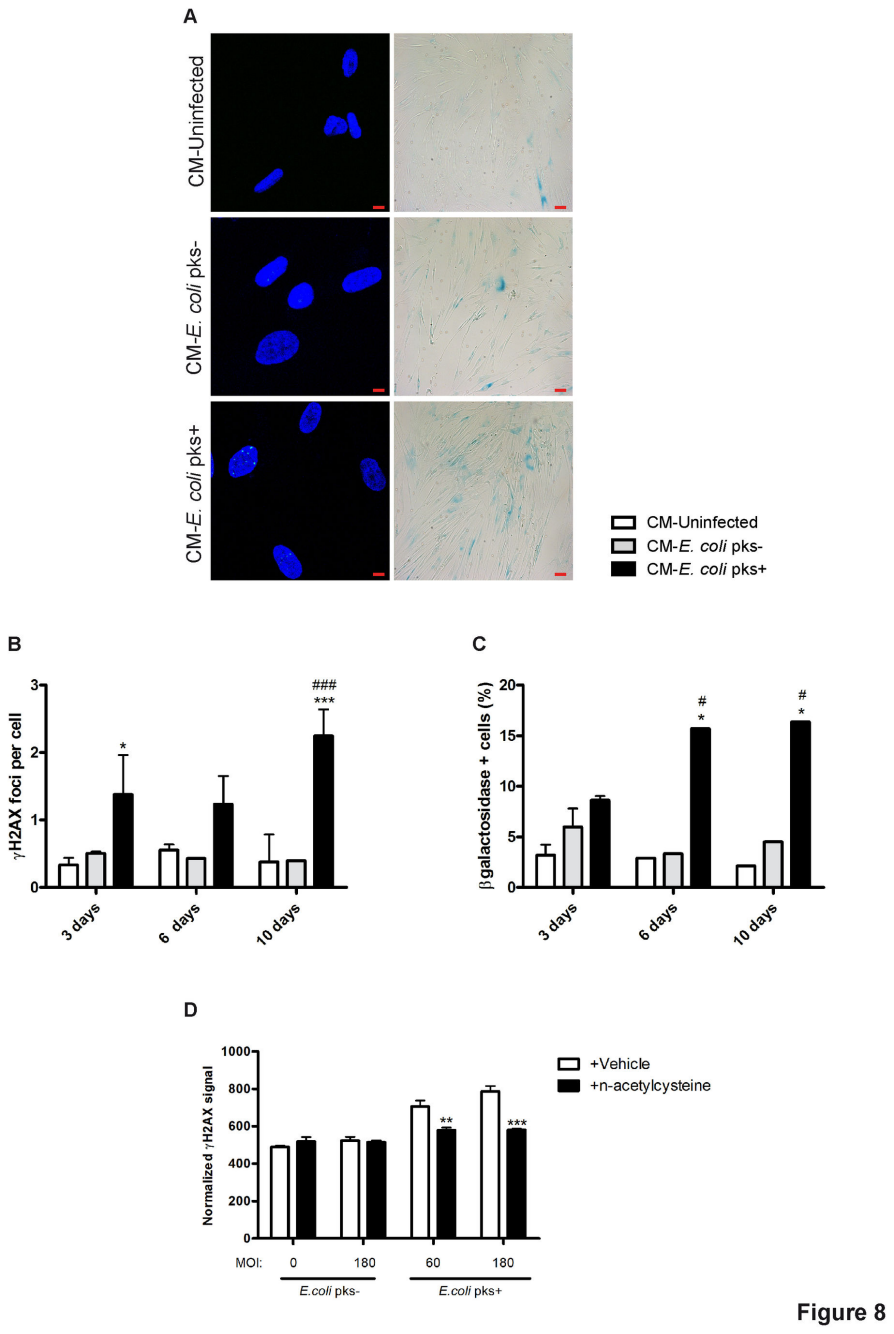
*Pks+ E. coli* infected IMR-90 cells induce bystander γH2AX foci formation and SA-β-Gal expression in uninfected IMR-90 cells. Naïve IMR-90 cells were treated for 1 day (γH2AX) or 3 days (SA-β-Gal) with conditioned media (CM) prepared 3-6-10 days after infection with *pks*+ or *pks*- *E*. *coli* with a MOI 180 (A, left panel). Cells were treated for 24 h with a 6 days-old CM and examined for DNA (Blue) and γH2AX (green), scale bars = 10µm (A, right panel). Cells were treated for 3 days with a 6 days old CM and examined for SA-β-Gal staining (Blue) (x20 magnification) (B) Numbers of γH2AX foci per cell (B) and percentages of SA-β-Gal positive cells (C) were quantified as before, 30-100 nuclei or 100-200 cells were evaluated for each condition. Results represent the mean and SEM of two independent experiments, one-way ANOVA with Bonferroni’s multiple comparison test; *P<0.05, ***P<0.001 comparing infected and uninfected groups; #P<0.05, # # #P<0.001 comparing *pks*+ and *pks*- groups. (D) IMR-90 grown on Transwells, were infected for 4h with live *pks*+ or *pks*- *E*. *coli*, washed and grown with gentamicin for 3 days. The Transwells were then transferred on top of naive IEC-6 cells and incubated for 24 hours with or without n-acetylcysteine (1mM). The IEC-6 cells were fixed and γH2AX was quantified by In-Cell Western. Results represent the mean and SEM of one experiments including triplicates for each conditions, two-way ANOVA with Bonferroni’s multiple comparison test; **P<0.01, ***P<0.001 comparing vehicle and n-acetylcysteine groups.

We next investigated whether the secretory phenotype observed in cells infected with *pks*+ *E. coli* could induce a “bystander” effect in neighbouring cells. Indeed, recent findings have shown that cytokine signalling pathways induced in drug-evoked senescence can drive senescence in neighbouring cells through autocrine or paracrine effects [37]. We harvested and filtered supernatant from IMR-90 at different time-points after infection. These conditioned media (CM) were used to treat naïve IMR-90 cells for one or three days and then we examined respectively the formation of γH2AX foci and expression of SA-β-Gal. We observed that the CM from *pks*+ *E. coli* infected IMR-90 cells induced γH2AX foci in recipient cells (Figure 8A, left panel). Similarly, the CM from IEC-6 cells infected with *pks*+ *E. coli* triggered γH2AX foci in naive cells (Figure S2). This bystander genotoxic effect increased slightly with the post-infection incubation duration of the infected cells (Figure 8B). This was accompanied with an increase of SA-β-Gal expression in recipient cells treated with CM from *pks*+ *E. coli* infected cells as compared to controls (Figure 8A, right panel). The percentages of CM-treated SA-β-Gal+ cells also increased with the post-infection incubation duration of the infected cells (Figure 8C). One of the major hypotheses accounting for the induction of DNA damage in recipient cells neighbouring senescent cells is the generation of ROS. We infected IMR-90 fibroblasts on Transwells and three days after the infection, we transferred these Transwells on sub-confluent naive IEC-6 cells and examined the formation of γH2AX foci in the presence or absence of the anti-oxidant n-acetylcysteine. We observed that *pks*+ *E. coli* infected IMR-90 cells enhanced γH2AX foci formation in underlying IEC-6 cells. This bystander effect was significantly reduced in the presence of n-acetylcysteine (Figure 8D). Therefore, the modification of the secretory expression profile and increased ROS production of the cells induced after infection with *pks*+ *E. coli* could be transmitted to bystander cells through a paracrine pathway.

### Colibactin-induced senescence promotes tumour cell growth in vitro

**Figure 9:**
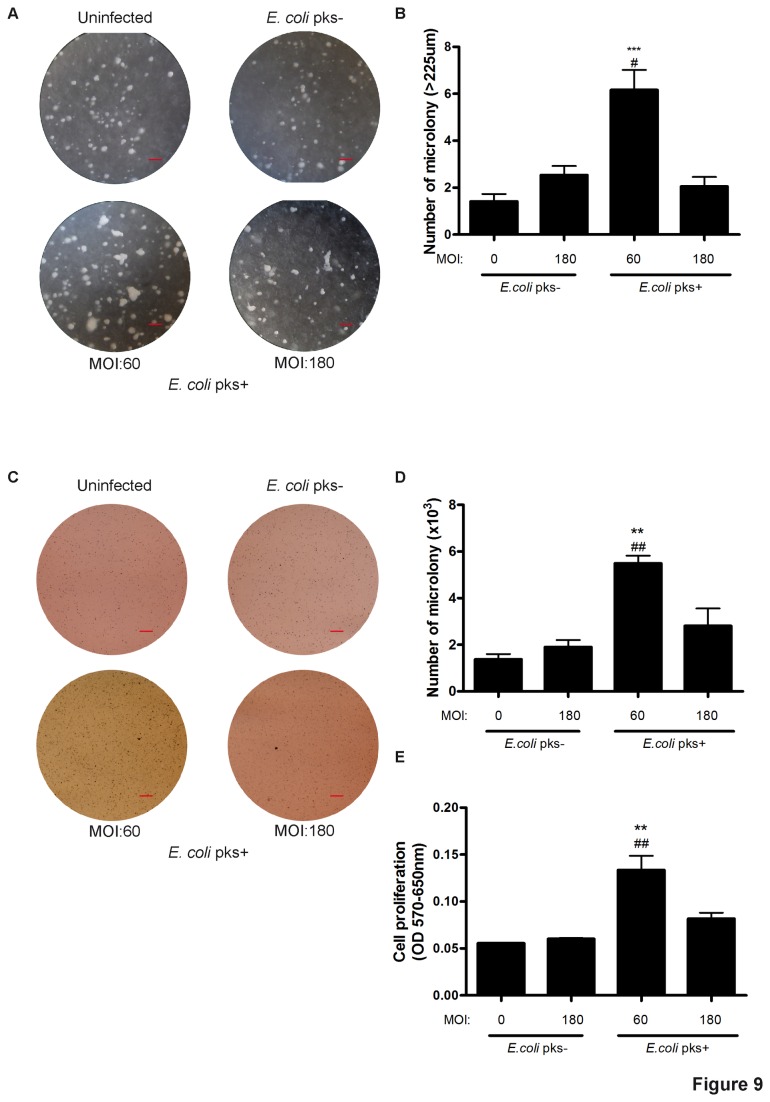
Senescent *pks+ E. coli* infected IMR-90 cells promote the growth of bystander tumour cells. IMR-90 cells were infected for 4h with live *pks*+ or *pks*- *E*. *coli* with an MOI of 60 or 180 or left uninfected. At the end of the infection, the cells were washed and grown with gentamicin for 3 days. Then the IMR-90 cells were layered with 0.5% agar and 5000 HCT116 p53^-/-^ cells embedded in 0.35% agar were added on top of the first layer and co-cultured for 10 days. (A) Representative photomicrographs of HCT116 p53^-/-^ cell colonies, scale bars = 0.7 mm. (B) Large (>225 µm) microcolonies per microscopy field were counted by a blinded observer. The upper HCT116 p53^-/-^ cell layer was harvested and stained with MTT for 24h. (C) Representative photomicrograph of experimental 6-well culture plates with viable cancer cell colonies coloured by MTT, scale bars = 3 mm. (D) The number of microcolonies was quantified using Image-J. (E) Total cell proliferation was assessed using MTT. Results represent the mean and SEM of two independent experiments, one-way ANOVA with Bonferroni’s multiple comparison test; *P<0.05 comparing infected and uninfected groups; #P<0.05 comparing *pks*+ and *pks*- groups.

Senescent stromal cells have been shown to support the growth of tumour cells, through soluble factors and extracellular matrix associated factors [36,38]. As we observed senescence and extracellular production of MMP-3 in IMR-90 cells following infection with *pks*+ *E. coli*, we next analysed whether these senescent fibroblasts could promote tumour cell growth using an *in vitro* co-culture system previously described [24]. A549 human lung adenocarcinoma cells were seeded with the IMR-90 cells three days after infection with *E. coli* producing or not colibactin, and co-cultured for 14 days. A549 colonies growth was demonstrated with Rhodanile blue staining (that preferentially stains cytokeratin-positive epithelial cell colonies) (Figure S3A). Stained surface analysis revealed that tumour cells co-cultured with fibroblasts previously infected with *pks*+ *E. coli* at MOI 60 shown enhanced growth that was statistically significant compared to *pks*- *E. coli* and uninfected controls (Figure S3B). In contrast, fibroblast infected with the highest MOI of *pks+ E. coli* did not increase cancer cell proliferation. In order to discriminate between a cell-contact and a paracrine effect, we performed Transwell experiments in which we incubated senescent IMR-90 fibroblasts over A549 cells for 5 days. We observed that A549 cell proliferation was enhanced when cultivated under MOI 60 *pks*+ *E. coli* infected fibroblasts (Figure S3C), in a similar manner to that observed in the Rhodanile Blue assay. To further examine whether *pks*+ *E. coli* infected IMR-90 cells could propel bystander tumour cell growth, we layered human colon carcinoma HCT116 p53^-/-^ cells in soft agar on top of infected IMR-90 cells. Using this soft agar colony formation system, we compared the effects of *pks*+ and *pks*- infected fibroblasts on the HCT116 p53^-/-^ cancer cell colony formation. *pks*+ IMR-90 were able to significantly support the formation of large HCT116 p53^-/-^ colonies as compared to *pks*- or uninfected fibroblasts (Figure 9A and B). MTT analysis of HCT116 p53^-/-^ colonies revealed that both colony multiplicity (Figure 9C and D) and cellular viability (Figure 9E) were enhanced when seeded on top of *pks*+ infected fibroblasts. Again, fibroblasts infected with the highest MOI of *pks+ E. coli* exhibited reduced potency to increase cancer cell proliferation. Taken together, these data demonstrated that the cellular senescence induced by the infection with *pks*+ *E. coli* could contribute to an acceleration of tumour cell growth in a paracrine manner.

## Discussion

In this study, we demonstrated that transient infection of non-transformed human cells with colibactin-producing *E. coli* induces premature cellular senescence. This senescence was associated with a cellular reprogramming characterized by the production of ROS and pro-inflammatory mediators. The secretory phenotype observed in cells infected with *pks*+ *E. coli* induced a “bystander” effect in neighbouring cells that could support for an increased tumour cell growth.

Cellular senescence was thought to result from an eroded-telomere dependent cell-cycle arrest, in order to prevent damaged DNA to be replicated and transmitted to the next generations [39]. However, a rich literature is now emerging showing that a diverse range of stimuli induces premature senescence [3]. Thus cellular senescence is now regarded as a complex phenomenon incorporating both genetic and environmental factors acting through convergent pathways. Nevertheless, only a limited number of studies highlights a role of bacterial products in the induction of cellular senescence while the role of senescence in the host-repair response to infection/inflammation is beginning to emerge [4]. Only two studies using respectively purified cytolethal distending toxin from *Haemophilus ducreyi* [40] or purified pyocyanin from *Pseudomonas aeruginosa* [41] have given preliminary evidences of the involvement of bacterial-derived toxins in the onset of senescence. Therefore, we asked whether infection of human cell-lines with *E. coli* strain producing the toxin colibactin could induce cellular senescence. Colibactin was known to induce acute DSB, cell cycle arrest and megalocytosis in eukaryotic cells [13]. Recently, we demonstrated that the DNA damages triggered upon low-dose infection with *E. coli* producing colibactin were frequently misrepaired leading to chronic chromosomal aberrations and increased gene mutation frequency [15]. Here we confirmed and extended these results by showing that human cells transiently infected with *E. coli* producing colibactin exhibited increased and persistent γH2AX foci, indicating DSB that were not repaired even after an extended period after the end of the infection. The activation of the DDR signalling pathway in fibroblasts after the infection with colibactin-producing *E. coli* was associated with a lack of S-phase and prolonged expression of p16^INK4^ and p21^CIP1^ concomitant with a decreased expression of pRb protein (data not shown). These CKI are critical regulators of cellular senescence through inactivation of pRb [42,43]. Most senescent cells express p16^INK4^, which on the contrary is rarely expressed by quiescent or terminally differentiated cells [44,45]. In addition, persistent activation of DDR signalling is now commonly regarded as the mechanism triggering cellular senescence [6], suggesting that infection with *E. coli* producing colibactin could induce cellular senescence. Indeed, we observed an increased expression of the SA-β-Gal, which is a classical feature of senescent cells [8,46] correlated with a nucleus enlargement which was previously reported in eukaryotic cells exposed to colibactin [13] and senescent cells [1]. Based on these data, we propose that the megalocytosis of colibactin-intoxicated cells associated with the persistent activation of the DDR, the cell-cycle arrest and the upregulation of SA-β-Gal expression reflects premature cellular senescence.

p16^INK4^ expression may orchestrate the formation of detectable senescent-associated heterochromatin foci (SAHF) [11,47]. Heterochromatin formation, characterized by the increased methylation of histone H3 on Lys9 (H3Me) or the presence of PML-nuclear bodies [29,31], has been considered critical for the induction of senescence, as heterochromatin was responsible for silencing proliferative genes [11,48]. In the present study, we observed in fibroblasts exposed to colibactin an increased formation of SAHF characterized by the presence of H3Me, in parallel with an augmented number of nuclear PML. Thus, human cell infected with *E. coli* producing colibactin encountered global nuclear reorganization that occurs at the onset of senescence and persists.

It is speculated that chromatin modification contributes to the activation and the regulation of gene expression modulation in response to genotoxic stress through the modification of specific histone methyltransferase [49]. Indeed, senescent cells are metabolically active and it has been recently established that they undergo a specific change in their protein expression and secretion program. This phenotype has been termed SASP [36] and is characterized by the secretion of 40–80 factors. Among these, senescent cells have been shown to release nitric oxide and reactive oxygen species due to alterations in inducible/endothelial nitric oxide synthase, superoxide dismutase or mitochondrial respiratory chain activities [50,51]. Passos et al proposed a causative link between DNA-damage, mitochondria-derived ROS and the onset of senescence [52]. In our study, we observed in fibroblasts a strong induction of intracellular and mitochondrial-derived ROS three and six days after the end of the infection with *E. coli* producing colibactin, when the phenotype of cellular senescence is plainly established. Senescent cells secrete also interleukins, inflammatory soluble mediators, and growth factors that can affect surrounding cells. Indeed, we observed a large production of both IL-6 and MMP-3 in the supernatant of fibroblasts infected with *E. coli* producing colibactin as compared to controls. IL-6 is the most prominent cytokine associated with DNA-damage or oncogenic-induced SASP in numerous human cell lines including fibroblasts [53,54]. Furthermore, IL-6 production appears to be directly under the control of a persistent DDR [9] and have an indispensable role in the establishment and maintenance of the senescence arrest [55]. The main MMP family members that have been consistently associated with human and mouse fibroblasts undergoing stress-induced senescence are stromelysin-1 and 2 (respectively MMP-3 and 10) [56,57]. As a result of these senescence-induced modifications in their secretory profile, senescent cells may influence their local microenvironment. This bystander phenomenon that have been broadly described in cells treated with ionizing radiation [58], is not well-known in the case of cellular senescence [59]. We observed that conditioned-media (containing SAPS mediators) from post-infected senescent IMR-90 or IEC-6 cells were able to induce both DNA damage and expression of SA-β-Gal in naïve recipient cells. Indeed, our data indicated that senescent cells induce a bystander effect that spreads DNA damage and SA-β-Gal in bystander cells. Interestingly, another intestinal human commensal bacterium, *Enterococcus faecalis* (*E. faecalis*), that presents a unique capacity in generating extracellular superoxide, induces both DNA damage and chromosomal instability in macrophages through a bystander effect [60]. Several determinants have been implicated in the development of this bystander effect, including Cox2-driven 4-hydroxy-2-nonenal production [61,62] or TNFα secretion [63]. These genomic damages induced by *E. faecalis* provide evidence that links bystander effect to the development of cancer, as colonized IL-10 knockout mice are susceptible to colorectal cancer [60]. Together, these data suggest that pro-oxidant and pro-inflammatory signals from primary or secondary senescent cells may induce deleterious changes in tissue microenvironment thus explaining the emerging roles of cellular senescence in tumour pathogenesis and aging [64].

Interestingly, cellular senescence seems to play opposite roles: as a tumour-suppressor mechanism when DNA-damage checkpoints are intact [42] or as inducer of genomic instability when DNA damage checkpoints are dysfunctional [22]. Several lines of evidence suggest that chronic exposure to infectious agents can cause telomere shortening, senescence and predisposition to aging and cancer. Indeed, studies have shown that patients suffering from chronic viral infections have short telomeres in specific T lymphocytes [65,66], elderly exhibited increased cancer prevalence and associated mortality [9] and shorter telomere in white blood cells correlated with increased mortality due to infectious diseases [59,67]. Furthermore, cancer is among the pathologies fuelled by inflammation [68]. Thus, SASP pro-inflammatory mediators may contribute to the development of tumours. In coculture experiments, we observed that fibroblasts infected with *E. coli* producing colibactin were able to promote the growth of epithelial tumour cells. This suggests that *pks+ E. coli* infection have the ability to instruct fibroblasts to reprogram their expression profiles to support cancer cell growth. To directly address this possibility, we used a soft agar microcolony system to co-culture infected fibroblast with colon cancer cells. We demonstrated that *pks+ E. coli* infected fibroblasts were able to promote cancer cell proliferation and expansion.

In addition, we demonstrated that SASP includes factors that are known to be associated with aggressive cancer cells [12,54], including IL-6 and MMP which can induce epithelial cell invasion or direct tumour promoting effects respectively [56]. The discrepancy we observed at the highest MOI of *pks+ E. coli* may suggest that the composition of the SASP induced in this specific condition may include toxic components and/or reduced levels of promoting factors that explain the decreased formation of cancer cell colonies. Taken together, these data provide insights into the cellular mechanisms leading to the pro-tumorigenic role of colibactin-producing *E. coli* in an experimental model of inflammation related colorectal cancer that was recently shown [69].

In conclusion, our study provides novel insights into the late cellular effects of colibactin intoxication. We established that the expression of the polyketide/non ribosomal peptide colibactin by *E. coli* was sufficient to induce in infected cells cellular senescence together with a metabolic reprogramming. In addition, our data are fully compatible with the idea that the production of pro-oxidant and pro-inflammatory mediators by senescent cells is the proximal cause of DNA damage in the bystander cells as well as a potent pro-tumorigenic actor.

## Supporting Information

Figure S1 **Increased PML nuclear bodies and SA-β-Gal expression in IEC-6 cells infected with *pks*+ *E. coli*.** Non-transformed rat intestinal epithelial IEC-6 cells were infected for 4h with live *pks*+ or *pks*- *E. coli* with an MOI of 20 and 100 bacteria per cell or left uninfected. At the end of the infection, the cells were washed and grown with gentamicin. (A) Cells were examined for DNA (blue) and PML protein (green) 2 or 6 days after infection. Pictures of uninfected and MOI 180-infected cells are shown, scale bars = 10µm(B) PML foci in 30-100 nuclei for each condition were counted by a blinded observer, in two independent experiments. (C) 6 days after infection, IMR-90 cells were fixed with formaldehyde 4% and then stained with X-gal blue for 24h. Percentage of SA-β-Gal positive cells was quantified. Results represent the mean and SEM of three pooled independent experiments, one-way ANOVA with Bonferroni’s multiple comparison test; **P<0.01, ***P<0.001 comparing infected and uninfected groups; # #P<0.01, # # #P<0.001 comparing *pks*+ and *pks*- groups, 100-200 cells were evaluated for each condition.
(TIF)

Figure S2 ***Pks+ E. coli* infected IEC-6 cells induce bystander γH2AX foci formation and SA-β-Gal expression in uninfected IEC-6 cells.** Naïve IEC-6 cells were treated for 1 day with CM prepared 3-6-14 days after infection with *pks*+ or *pks*- *E. coli* with a MOI 180. (A) Cells were examined for DNA (Blue) and γH2AX (green) 1 day treatment with CM (Scale bars = 10µm) (B) Numbers of γH2AX foci per cell were quantified, 50-100 nuclei were evaluated for each condition.
(TIF)

Figure S3 **Senescent *pks*** ***+ E. coli* infected IMR-90 cells promote the growth of bystander A-549** and HCT-116 p53^-/-^ **tumour cells**.
IMR-90 cells were infected for 4h with live *pks*+ or *pks*- *E. coli* with an MOI of 20, 60 or 180 or left uninfected. At the end of the infection, the cells were washed and grown with gentamicin for 3 days. Then, 5000 A549 cells were plated on top of IMR-90 and co-cultured for 15 days in 1% serum medium. Cells were fixed with 4% formaldehyde and stained with 1% Rhodanile Blue that stains preferentially A549 cells. (A) Representative scanned photomicrograph of experimental 6-wells culture plate. (B) The Rhodanile Blue stained area was quantified in each well using Image-J in the green channel extracted from the RGB photomicrographs. (C) IMR-90 cells grown on Transwells were infected for 4h with live *pks*+ or *pks*- *E. coli* with an MOI of 60 or 180 or left uninfected. At the end of the infection, the cells were washed and grown with gentamicin for 3 days. The transwells were then transferred on top of 5000 HCT-116 p53^-/-^ cells and incubated for 5 days. Cancer cell proliferation was assessed using MTT. Results represent the mean and SEM of three independent experiments, one-way ANOVA with Bonferroni’s multiple comparison test; *P<0.05 comparing infected and uninfected groups; #P<0.05 comparing *pks*+ and *pks*- groups.
(TIF)
