# Supplementary figures and images for: Escherichia coli Producing Colibactin Triggers Premature and Transmissible Senescence in Mammalian Cells
Source: PLoS One. 2013 Oct 8;8(10):e77157. doi: 10.1371/journal.pone.0077157 (PMC3792898; doi:10.1371/journal.pone.0077157)

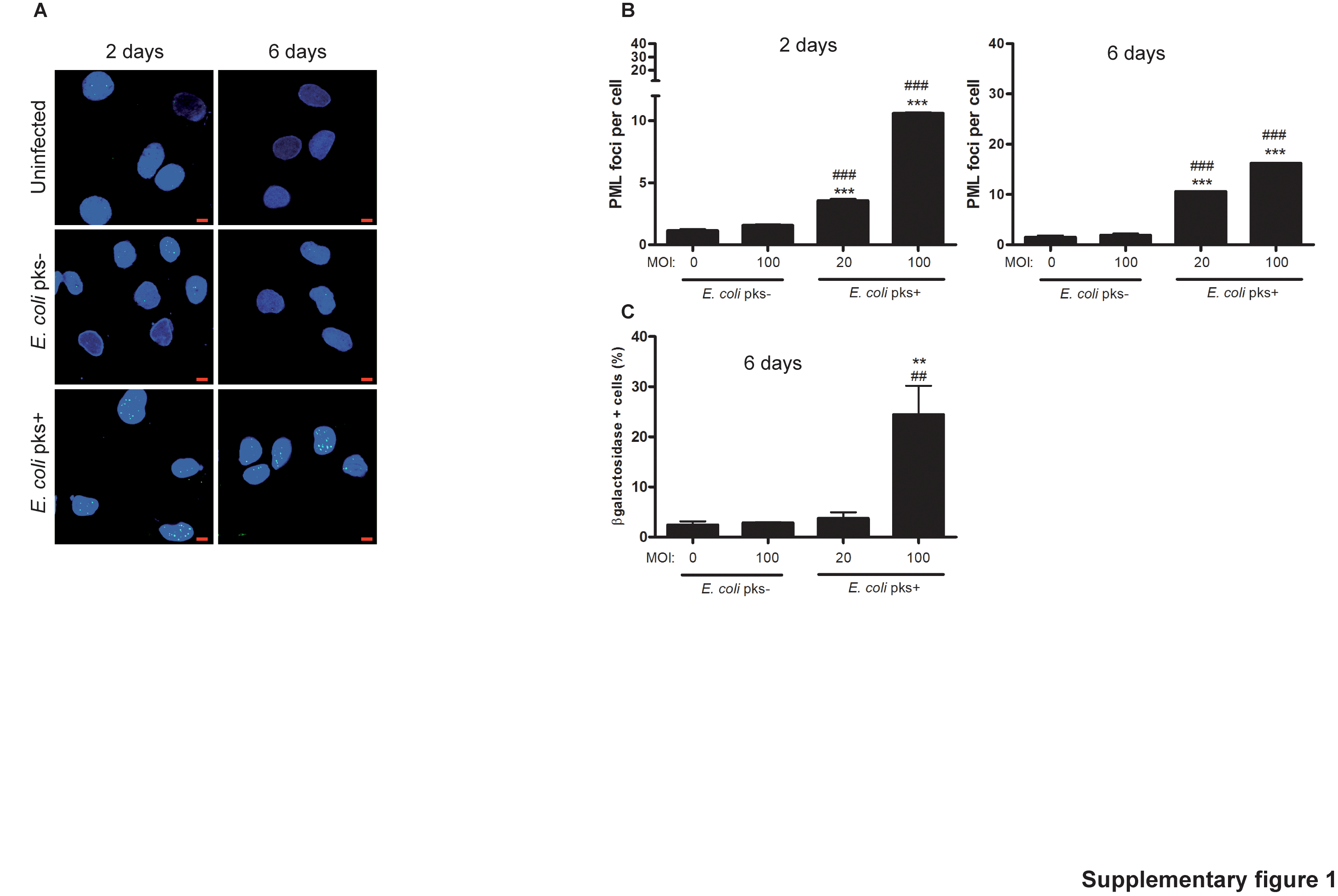

Supplement: Figure S1 — Increased PML nuclear bodies and SA-β-Gal expression in IEC-6 cells infected with pks+ E. coli. Non-transformed rat intestinal epithelial IEC-6 cells were infected for 4h with live pks+ or pks- E. coli with an MOI of 20 and 100 bacteria per cell or left uninfected. At the end of the infection, the cells were washed and grown with gentamicin. (A) Cells were examined for DNA (blue) and PML protein (green) 2 or 6 days after infection. Pictures of uninfected and MOI 180-infected cells are shown, scale bars = 10µm(B) PML foci in 30-100 nuclei for each condition were counted by a blinded observer, in two independent experiments. (C) 6 days after infection, IMR-90 cells were fixed with formaldehyde 4% and then stained with X-gal blue for 24h. Percentage of SA-β-Gal positive cells was quantified. Results represent the mean and SEM of three pooled independent experiments, one-way ANOVA with Bonferroni’s multiple comparison test; **P<0.01, ***P<0.001 comparing infected and uninfected groups; # #P<0.01, # # #P<0.001 comparing pks+ and pks- groups, 100-200 cells were evaluated for each condition. (TIF) [file pone.0077157.s001.tif]

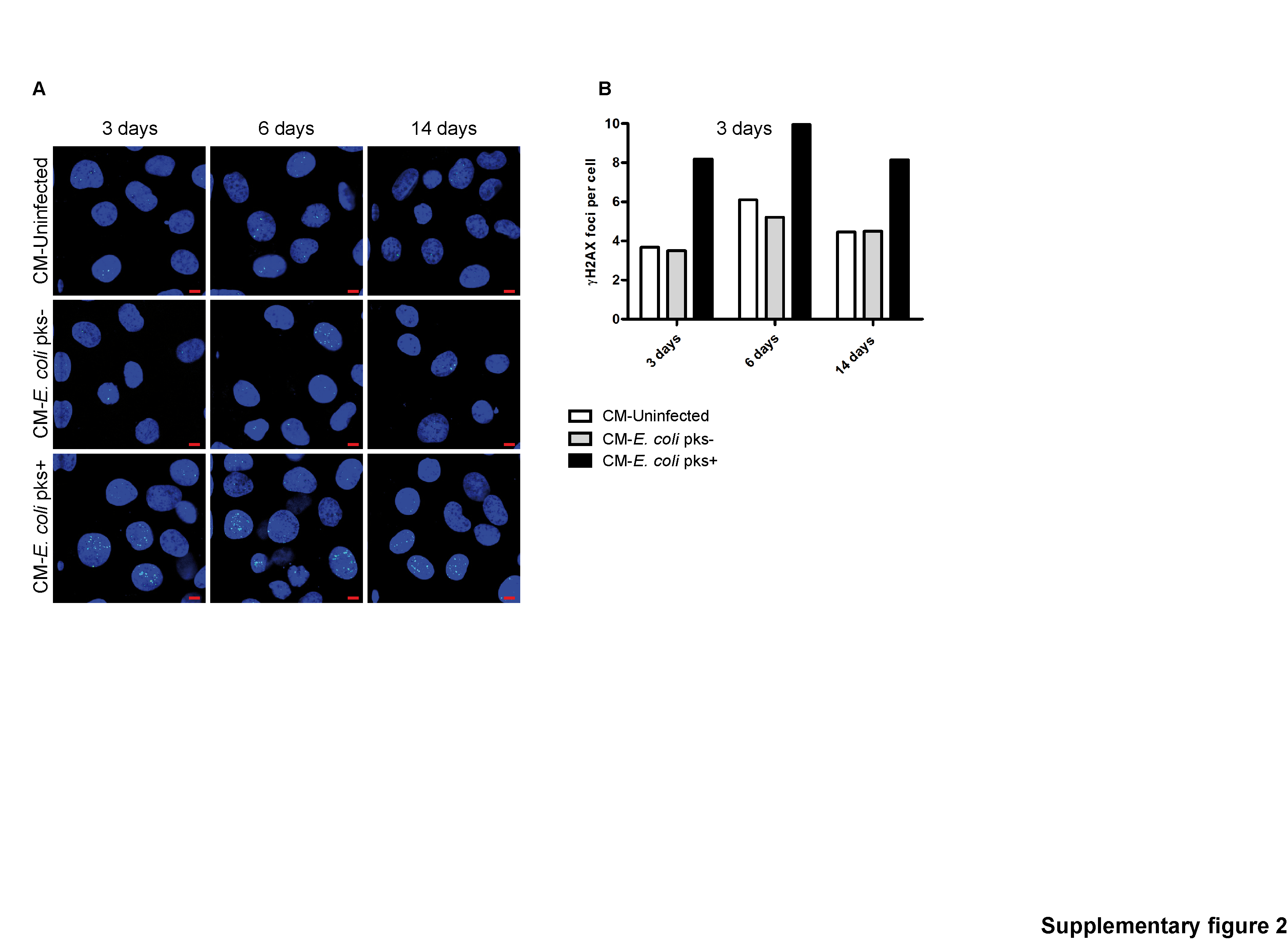

Supplement: Figure S2 — Pks+ E. coli infected IEC-6 cells induce bystander γH2AX foci formation and SA-β-Gal expression in uninfected IEC-6 cells. Naïve IEC-6 cells were treated for 1 day with CM prepared 3-6-14 days after infection with pks+ or pks- E. coli with a MOI 180. (A) Cells were examined for DNA (Blue) and γH2AX (green) 1 day treatment with CM (Scale bars = 10µm) (B) Numbers of γH2AX foci per cell were quantified, 50-100 nuclei were evaluated for each condition. (TIF) [file pone.0077157.s002.tif]

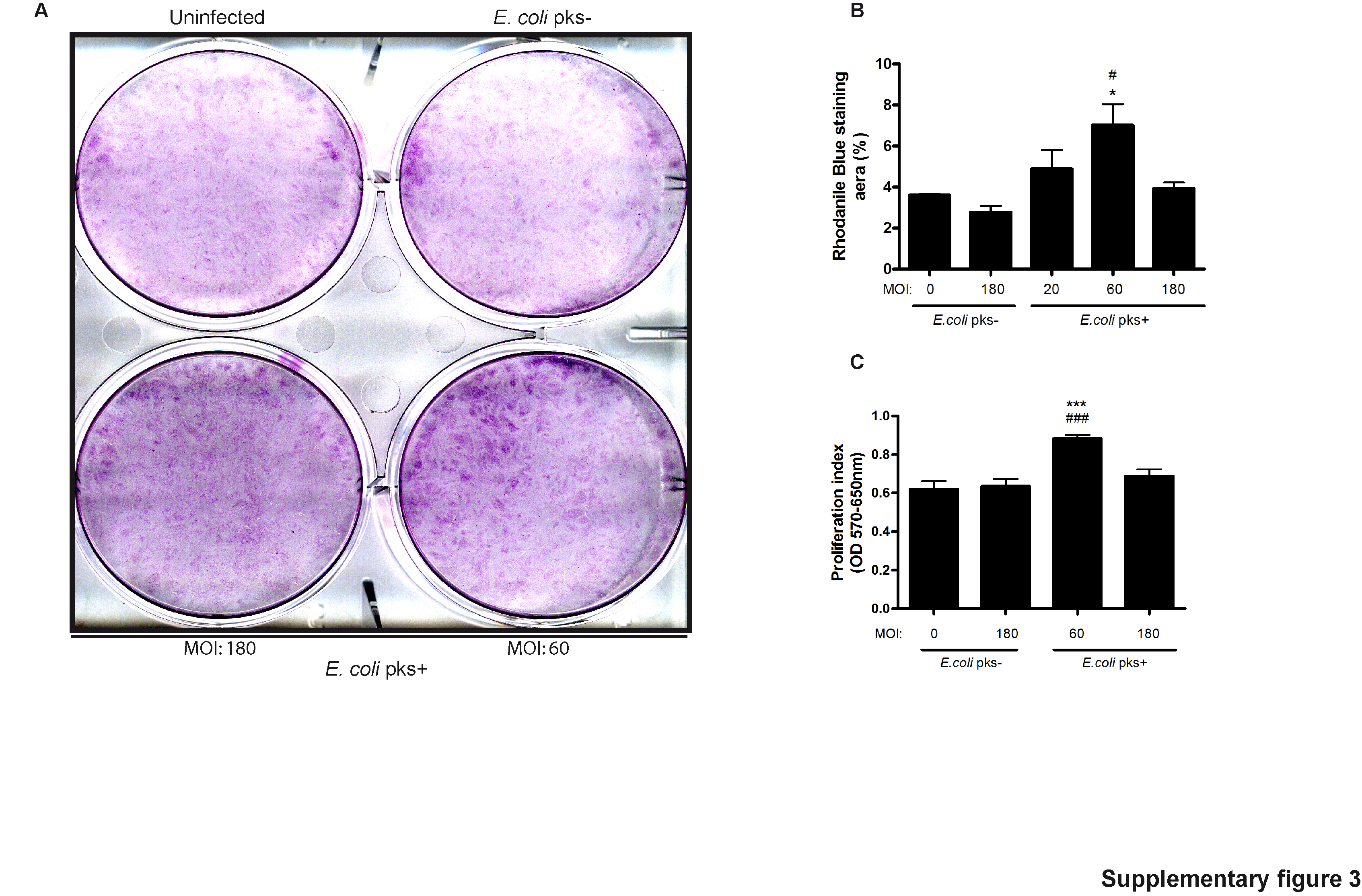

Supplement: Figure S3 — Senescent pks + E. coli infected IMR-90 cells promote the growth of bystander A-549 and HCT-116 p53-/- tumour cells. IMR-90 cells were infected for 4h with live pks+ or pks- E. coli with an MOI of 20, 60 or 180 or left uninfected. At the end of the infection, the cells were washed and grown with gentamicin for 3 days. Then, 5000 A549 cells were plated on top of IMR-90 and co-cultured for 15 days in 1% serum medium. Cells were fixed with 4% formaldehyde and stained with 1% Rhodanile Blue that stains preferentially A549 cells. (A) Representative scanned photomicrograph of experimental 6-wells culture plate. (B) The Rhodanile Blue stained area was quantified in each well using Image-J in the green channel extracted from the RGB photomicrographs. (C) IMR-90 cells grown on Transwells were infected for 4h with live pks+ or pks- E. coli with an MOI of 60 or 180 or left uninfected. At the end of the infection, the cells were washed and grown with gentamicin for 3 days. The transwells were then transferred on top of 5000 HCT-116 p53-/- cells and incubated for 5 days. Cancer cell proliferation was assessed using MTT. Results represent the mean and SEM of three independent experiments, one-way ANOVA with Bonferroni’s multiple comparison test; *P<0.05 comparing infected and uninfected groups; #P<0.05 comparing pks+ and pks- groups. (TIF) [file pone.0077157.s003.tif]
